# Alkali Source Modulates Polydopamine Shell Thickness and Ferroptosis Susceptibility in Iron Oxide Nanoparticles Under Magnetic Hyperthermia

**DOI:** 10.1155/bca/8031913

**Published:** 2026-09-26

**Authors:** Kübra Solak, Yagmur Unver, Ahmet Mavi

**Affiliations:** ^1^ East Anatolia High Technology Application and Research Center (DAYTAM), Atatürk University, 25240, Erzurum, Türkiye, atauni.edu.tr; ^2^ Department of Molecular Biology and Genetics, Faculty of Science, Atatürk University, Erzurum, Türkiye, atauni.edu.tr; ^3^ Department of Molecular Biology and Genetics, Faculty of Science, Erzurum Technical University, Erzurum, Türkiye, erzurum.edu.tr; ^4^ Department of Nanoscience and Nanoengineering, Institute of Science and Technology, Atatürk University, Erzurum, Türkiye, atauni.edu.tr; ^5^ Department of Mathematics and Science Education, Education Faculty of Kazim Karabekir, Atatürk University, Erzurum, Türkiye, atauni.edu.tr

**Keywords:** ferroptosis, magnetic hyperthermia, one-pot synthesis, oxidative stress, polydopamine-coated magnetic nanoparticles

## Abstract

Controlling nanoparticle surface chemistry through synthesis conditions—rather than postsynthetic modification—represents an attractive strategy for tailoring therapeutic behavior. Here, we report that the choice of alkali source during one‐pot co‐precipitation of polydopamine‐coated iron oxide magnetic nanoparticles (PDA‐Fe_3_O_4_ MNPs) determine their physicochemical identity and downstream biological activity. The type of base influenced particle properties: NaOH (MNP1) produced smaller (9.4 nm), well‐crystallized particles with a negative PDA shell (*ζ* = −25.0 mV), whereas ammonia (MNP2) yielded larger particles (11.2 nm) with a higher positive surface (*ζ* = +45.5 mV) charge, which was associated with greater cellular uptake. In HUVEC and SH‐SY5Y cells, MNPs were biocompatible at low concentrations. In SH‐SY5Y cells, when MNP1 was applied with magnetic hyperthermia (MHT+), ROS levels increased to 38.6%, while the lipid peroxidation MFI red/green ratio decreased from ~65 to ~58, indicating a marked ferroptosis tendency. Conversely, MNP2 caused greater membrane damage, with lower ROS production under MHT+ (12.4%) compare to MNP1 (38.6%) and a smaller decline in the lipid peroxidation ratio (from ~60 under MHT‐ to ~55 under MHT+) in SH‐SY5Y. Notably, MNP2’s own ROS levels decreased from MHT‐ (22.8%) to MHT+ (12.4%), the opposite trend observed for MNP1. Ferroptosis‐related cell death was not observed in HUVECs, as assessed by ROS and lipid peroxidation assays. Furthermore, the 5.2‐fold increase in *FTH1* gene expression in SH‐SY5Y cells following MNP1 treatment suggests that the cells activate intracellular iron buffering as a bioinorganic defense mechanism and that different cell death pathways associated with ferroptosis may be triggered depending on the type of nanoparticle. Critically, the formulation induced neither significant cytotoxicity nor markers of ferroptosis in HUVECs, suggesting a degree of cancer‐cell selectivity for MNPs. These findings establish that base‐dependent surface engineering of PDA‐Fe_3_O_4_ MNPs yield functionally distinct nanoplatforms and provide a rational foundation for the design of ferroptosis‐enhanced magnetothermal cancer therapies.

## 1. Introduction

Hyperthermia has emerged as an alternative supporting therapy in the treatment of cancer. It involves controlled heating of tissues or cells to 39°C–45°C, typically achieved using radiofrequency waves, light, or magnetic fields [1]. These elevated temperatures directly induce cell death and enhance the sensitivity of tumor cells to radiotherapy or chemotherapy, potentially boosting their effectiveness by up to 10 times [2].

Magnetic nanoparticles (MNPs) are commonly used in hyperthermia applications to provide localized heating in response to an alternating magnetic field, thereby minimizing damage to surrounding healthy tissue. Inorganic nanoparticles—including superparamagnetic iron oxide (e.g., Dexferrum, Venofer), gold, manganese, and silica‐based NPs—have shown potential in tumor photothermal therapy, radiosensitization, and targeted drug delivery [3]. Despite their promise, concerns regarding safety, reproducibility, comprehensive cost–benefit analyses, and scalability persist, requiring further research on NPs. Surface modification of MNPs is commonly employed to improve their therapeutic performance. However, the reactions, precursors, and reaction conditions affect the size, distribution, crystal structure, and magnetic properties of MNPs [4–7].

Dopamine (DA) coating is often employed to enhance the stability, biocompatibility, and dispersibility of these MNPs owing to the presence of catechol and amine groups. Typically, DA coating of MNPs is performed as a postsynthesis step [8, 9]. The direct application of DA during synthesis (one‐pot production) is expected to streamline the manufacturing process and lower costs. In this case, depending on the base used (e.g., NaOH or ammonia), changes in the phenolic hydroxyl groups of DA or accelerated oxidation of DA under alkaline conditions may occur.

**SCHEME 1:**
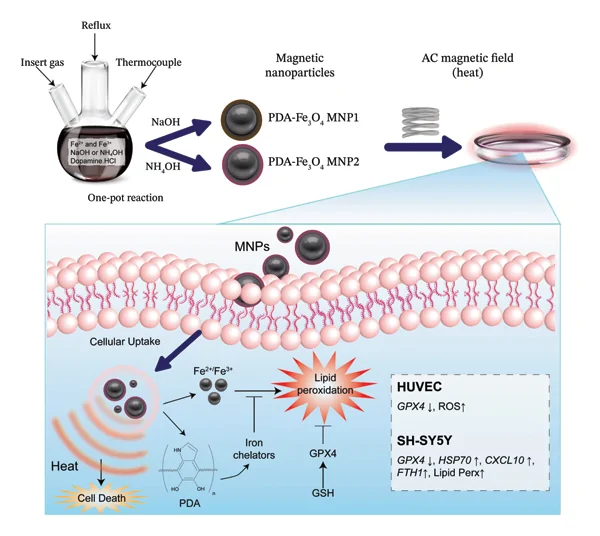
Schematic summary of this study. Polydopamine‐coated iron oxide magnetic nanoparticles (PDA‐Fe_3_O_4_ MNPs) synthesized with NaOH (MNP1) and NH_4_OH (MNP2) showed a high antitumor effect. Cellular stress, apoptosis, and reactive oxygen species (ROS) increase in SH‐SY5Y cells, triggering distinct stress responses, such as inflammation and lipid peroxidation, with limited toxicity in HUVECs. AC: alternating current, MHT: magnetic hyperthermia. GPX4: glutathione peroxidase 4, HSP70: heat shock protein 70 (Hsp70 or DnaK), CASP3: caspase‐3.

In this study, PDA‐coated Fe_3_O_4_ MNPs were synthesized via a one‐pot method using two different base sources (NaOH and ammonia). The central hypothesis of this study is that the base source determines PDA polymerization efficiency and, in turn, sequentially regulates the downstream biological responses of cells treated with these MNPs. This study aims to (i) synthesize polydopamine‐coated iron oxide magnetic nanoparticles (PDA‐Fe_3_O_4_) MNPs using a one‐step synthesis method via two different base sources (MNP1 with NaOH and MNP2 with NH_4_OH) and to elucidate the effects of the difference in base sources on the physicochemical properties of the nanoparticles; (ii) to understand the biological effects of these different synthesis conditions (effects on intracellular uptake, cytotoxicity, apoptosis, oxidative stress, lipid peroxidation, and gene expression); and (iii) to comparatively investigate MNP1 and MNP2 using both a cancer (SH‐SY5Y neuroblastoma) and a normal (HUVEC) cell model with or without magnetic hyperthermia (MHT) treatment. A schematic representation of the study is given in Scheme 1.

## 2. Material and Methods

### 2.1. Materials

All chemicals were purchased from Sigma‐Aldrich (USA). The following chemicals were used in the study: sodium hydroxide (NaOH), ammonia solution (25%–30%, NH_4_OH), dimethyl sulfoxide (DMSO), hydrochloric acid (HCl), iron (III) chloride hexahydrate (FeCl_3_·6H_2_O), iron (II) chloride tetrahydrate (FeCl_2_·4H_2_O), dopamine hydrochloride, hydrocortisone, and paraformaldehyde. Phosphate‐buffered saline (PBS), fetal bovine serum (FBS), Dulbecco’s modified Eagle medium (DMEM), antibiotic solution, and DMEM/Nutrient Mixture F‐12 (DMEM/F‐12) were obtained from Gibco, Thermo Fisher Scientific, Waltham, MA, USA. Epidermal growth factor (EGF) was purchased from PeproTech (Rocky Hill, NJ, USA).

The following kits were used in the study: MTS assay kit (ab197010, Abcam, Cambridge, UK), lipid peroxidation assay kit (ab243377, Abcam, Cambridge, UK), Annexin V‐FITC/PI apoptosis detection kit (640914, BioLegend, San Diego, CA, USA), RNA isolation kit (EcoPure, Ecotech Biotechnology, Erzurum, Türkiye), cDNA synthesis kit (SuScript RT01A025, Sugenomics Biotechnology, Ankara, Türkiye), and qPCR master mix (SuYBRGreen PCR01C0251, Sugenomics Biotechnology, Ankara, Türkiye).

### 2.2. Preparation of MNPs

The MNPs were produced with a modified surface by a one‐pot reaction, as described in our previous articles [10]. The sodium hydroxide solution (0.63 M, 29 mL) was boiled under a nitrogen atmosphere, and 1 mL of dopamine hydrochloride (0.03 mmol, dissolved in NaOH) was added to the reaction flask. Then, 1.35 g of FeCl_3_·6H_2_O (2.5 mL) and 0.5 g of FeCl_2_·4H_2_O (2.5 mL) were dissolved in ultra‐pure water before being injected into the reaction flask. The reaction mixture was then refluxed at 95°C for 2 h and cooled to room temperature. The Fe_3_O_4_@PDA MNP1 were collected using a magnet (Mıknatıs Dünyası, Türkiye), washed several times with water, dispersed in ultrapure water, and then stored at +4°C under nitrogen protection.

Fe_3_O_4_@PDA MNP2 were synthesized using ammonia as the base. 1.35 g of FeCl_3_·6H_2_O and 0.5 g of FeCl_2_·4H_2_O were dissolved in 29 mL of ultra‐pure water and then heated to boiling. Subsequently, 1 mL of dopamine hydrochloride (0.03 mmole, dissolved in water) and 5 mL of ammonia solution (25%–30%) were injected, respectively. After 2 h of reaction, the mixture was cooled to room temperature. The Fe_3_O_4_@PDA MNP2 were collected using a magnet, washed several times with water, dispersed in ultrapure water, and then stored at +4°C under nitrogen protection.

### 2.3. Characterization of MNPs

The synthesized MNPs were characterized using transmission electron microscopy (TEM, Hitachi HighTech HT7700, Tokyo, Japan), X‐ray diffraction (XRD, EMPYREAN, Malvern Panalytical, Almelo, Netherlands), Fourier‐transform infrared spectroscopy (FTIR, Bruker VERTEX 70v, Bruker, Billerica, MA, USA), and dynamic light scattering (DLS, ZS90, Malvern Instruments, Worcestershire, UK). All measurements and analyses were performed at DAYTAM (Eastern Anatolia High Technology Application and Research Center), Atatürk University, Erzurum, Türkiye.

TEM imaging provided detailed insights into the morphology and size distribution of the MNPs. Samples were placed on a carbon‐coated copper grid and air‐dried before examination at 120 kV. XRD analysis revealed the crystalline nature and phase composition of the MNPs, affirming the presence of iron oxide. It utilized Cu Kα radiation (*λ* = 1.5406 Å) over a 10°–90° range with 0.02° steps, using settings of 40 mA and 45 kV. A VERTEX 70v FTIR at 4 cm^−1^ resolution from 4000 to 400 cm^−1^ in ATR mode confirmed the chemical structure of MNPs. The zeta meter was used to measure the ζ‐potential, hydrodynamic radius (Rh), and polydispersity index (PDI) of the MNPs. Before measurement, the MNPs were diluted in water, and then the sample was loaded into a scattering cell at 25°C. Inductively coupled plasma (ICP, Agilent 7800, Agilent Technologies, Santa Clara, CA, USA) was utilized for elemental analysis and quantification of iron content. Samples were digested by microwave and acid before measurement. The specific absorption rate (SAR) of MNPs under alternating magnetic fields (MSI Automation, USA) was calculated using a constant iron concentration in the MNP solutions (1000 ppm Fe/mL), as described in our previous study [11].

### 2.4. Cell Culture

The SH‐SY5Y (RRID: CVCL_0019) and HUVEC (RRID: CVCL_2959) cell lines were gifted by Assoc. Prof. Ferhunde AYSIN, DAYTAM, Türkiye. The SH‐SY5Y cells were cultured in high‐glucose DMEM, supplemented with 10% FBS and 1% antibiotic solution. HUVECs were cultured in DMEM/F‐12 supplemented with 10% fetal bovine serum, 1% antibiotic, 0.5 μg/mL hydrocortisone, and 20 ng/mL EGF. The cells were placed in a NUAIRE incubator and incubated at 37°C, 5% CO_2_, and 90% humidity.

### 2.5. Prussian Blue Staining

Prussian blue staining, a histochemical method acceptable as an alternative to TEM, was used to examine the cellular uptake of MNP1 and MNP2. A total of 50,000 cells were seeded in 48‐well culture plates. After 12 h of incubation at 37°C, the culture medium was replaced with 0.75 mL of fresh medium containing 25, 50, or 100 μg/mL of MNPs. A group of cells was incubated on a magnet for 10 min. After 6 h, the cells were washed with ice‐cold PBS to remove free MNPs. The cells were treated with 2% paraformaldehyde for 20 min to immobilize them, followed by a 5‐min permeabilization process using Triton X‐100 (0.3%) at room temperature. The cells were exposed to a fresh Prussian blue solution, which was made by combining equal volumes of a 2% HCl solution and a 10% potassium ferrocyanide solution [12]. The incubation was carried out for 30 min at 37°C. The cells were washed with PBS and imaged by a reverse microscope (Carl Zeiss AG, Oberkochen, Germany) and a camera (Axiocam ERc 5s, Oberkochen, Germany).

### 2.6. Crystal Violet (CV) Assay

Cells were planted in 96‐well culture plates (7000 cells/well) and incubated for 24 h at 37°C. The culture medium was changed with 200 μL of fresh media containing 0–200 μg/mL of PDA‐Fe_3_O_4_ MNPs. A group of cells was incubated on a magnet for 10 min. After 24‐ and 48‐h incubation, the cells were rinsed once with phosphate‐buffered saline and then treated with 50 μL of a 0.5% CV solution (2.5 g of CV dissolved in a mixture of 100 mL methanol and 400 mL distilled water) [13]. After a 10‐min exposure, any excess CV was removed, and the wells were rinsed 3 times with tap water. The substance in the wells was dissolved in 200 μL of methanol and measured at 555 nm using a plate reader (Epoch, BioTek Instruments, Winooski, VT, USA).

### 2.7. MTS Assay

The cells were seeded in a 96‐well plate at a density of 10,000 cells per well and treated with PDA‐Fe_3_O_4_ MNPs after 24 h of incubation at 37°C. The cells were treated with different concentrations of PDA‐Fe_3_O_4_ MNP. The plates were placed in an incubator at 37°C for 10 min, with a magnet in place. After 48 h of treatment, 20 μL of MTS solution was added to the cells in the 96‐well plates (200 μL). After 2 hours of MTS addition, the absorbance was measured at 490 nm using a microplate reader (Epoch, BioTek Instruments, Winooski, VT, USA). The MTS assay (197010, Abcam) is a colorimetric method for sensitive quantification of viable cells after treatment. The vitality (%) of the treatment groups was determined by comparing the absorbance of the control group cells.

### 2.8. MHT

The cells were seeded in a 35 mm cell culture dish at a density of 350,000 cells per dish and incubated for 24 h at 37°C. The PDA‐Fe_3_O_4_ MNPs‐treated (50 μg/mL) groups were incubated on a magnet for 10 min at 37°C. After 6 h, the culture plate was placed in the MHT device (MSI automation) and subjected to an alternating magnetic field (250 kHz) for 3 h (temperature range: > 39°C to < 42°C), as performed in our previous studies [14, 15]. The treated cells were incubated for 48 h before conducting the apoptosis experiment.

### 2.9. Apoptosis Test

The PDA‐Fe_3_O_4_ MNPs and MHT‐treated cells were collected after 48 h by trypsinization and then suspended in Annexin V Binding Buffer. Then, 2.5 μL of FITC Annexin V and 5 μL of propidium iodide solutions were added to 100 μL of cell suspension (640914, Biolegend). The mixture was then well mixed using a pipette. The samples were placed in the dark at room temperature for 15 min and subsequently examined using flow cytometry (Cytexpert Beckman Coulter, Brea, California, United States) in Annexin V Binding Buffer. Nonstaining cells were used for gating, and a minimum of 15,000 events were recorded.

### 2.10. Cellular Reactive Oxygen Species (ROS) Assay

Intracellular ROS levels were determined using the commercial DCFDA/H_2_DCFDA Cellular ROS Assay Kit (ab113851, Abcam) in accordance with the manufacturer’s protocol. This method is based on the principle that the probe, which diffuses into the cell and is deacetylated by esterases to form 2′,7′‐dichlorofluorescin diacetate (DCFDA, also known as H_2_DCFDA, DCFH‐DA, and DCFH), is oxidized by ROS to the highly fluorescent 2′, 7′‐dichlorofluorescein (DCF) form. Cells treated with PDA‐Fe_3_O_4_ MNPs and MHT for 48 h were collected, washed with PBS, and incubated in the dark at 37°C in a serum‐free medium with the DCFDA working solution. Following incubation, the cells were washed again, and the fluorescence signal was measured by flow cytometry (Ex/Em ≈ 485/535 nm). Gating was performed using nonstaining cells, and at least 15,000 events were recorded.

### 2.11. Lipid Peroxidation Assay

The Lipid Peroxidation Assay Kit (ab243377, Abcam) utilizes a sensitive lipid peroxidation sensor that changes fluorescence from red to green upon exposure to ROS in cells. The red/green ratio decreases as ROS and lipid peroxidation levels increase. Here, cells treated with PDA‐Fe_3_O_4_ MNPs and MHT for 48 h were collected and suspended in Hank’s balanced salt solution (HBSS). The Lipid Peroxidation Sensor was applied to the cells at a final concentration of 1x according to the manufacturer’s protocol. The cells were cultured for 30 min at 37°C in a cell incubator with 5% CO_2_. Peroxidation was evaluated by flow cytometry after the cells were washed with HHBS. Gating was performed using nonstaining cells, and at least 15,000 events were recorded.

### 2.12. Quantitative Real‐Time PCR (qRT‐PCR)

The cells previously cultured in 25 cm^2^ culture dishes were treated with PDA‐Fe_3_O_4_ MNPs and MHT. The cells were then harvested using a trypsin‐EDTA solution, and RNA was isolated according to the protocol for the EcoPure RNA isolation kit (Ecotech Biotechnology, Erzurum, Türkiye). The concentration and purity of the isolated RNA were determined using a Nanodrop spectrophotometer (BioTek), and agarose gel electrophoresis (1.5% agarose, 1x TBE, 100 V for 30 min) was performed. The RNA samples were stored at −80°C for further use. All RNA samples were obtained at high concentration and purity.

For cDNA synthesis, 750 ng of RNA from each sample was mixed with nuclease‐free water to a final volume of 20 μL, and the mixture was placed into tubes containing 4 μL of cDNA synthesis mix (SuScript, RT01A025, Sugenomics Biotechnology, Ankara, Türkiye). The samples were incubated in a thermal cycler at 42°C for 1 h (reverse transcription reaction) and then at 80°C for 10 min (to inactivate reverse transcriptase). The resulting cDNAs were stored at −20°C until use.

qRT‐PCR was performed using 2X SuYBRGreen qPCR Mastermix (PCR01C0251, Sugenomics Biotechnology, Ankara, Türkiye), which contains HotStart polymerase. The manufacturer’s recommended protocol was followed for the PCR procedure, including HotStart activation at 95°C for 5 min, followed by 40 cycles of denaturation (95°C, 15 s), and annealing/extension (60°C, 60 s); melting curve from 60°C to 95°C at 0.1°C/s [16]. The target genes and primer sequences used for qRT‐PCR are provided in Table 1. *GAPDH* was used as the housekeeping (internal control) gene, and ΔΔCT values (*n* = 3), normalized to GAPDH, were used to evaluate and compare qRT‐PCR results [17].

**TABLE 1:** Primer sequences for the genes were taken from the literature and used in the study.

Gene (human)	Primers	Ref.
GAPDH	Forward, 5′‐GTCTCCTCTGACTTCAACAGCG‐3′	[18]
	Reverse, 5′‐ACCACCCTGTTGCTGTAGCCAA‐3′	

GPX4	Forward:5′‐AGAGATCAAAGAGTTCGCCGC‐3′	[19]
	Reverse: 5′‐TCTTCATCCACTTCCACA GCG‐3′	

HSP70	Forward: 5′‐TTTTACCACTGAGCAAGTGACTG‐3′	[20]
	Reverse: 5′‐ACAAGGAACCGAAACAACACA‐3′	

CASPASE 3	Forward: 5′‐CCAAAGATCATACATGGAAGCG‐3′	[21]
	Reverse: 5′‐CTGAATGTTTCCCTGAGGTTTG‐3′	

FTH1	Forward: 5′‐CAGACTGTGATGACTGGGAG‐3′	[22]
	Reverse:5′‐CTCAATGAAGTCACACAAATGGG‐3′	

CXCL10	Forward: 5′‐CCATTCTGATTTGCTGCCTTATC‐3′	[23]
	Reverse:5′‐TACTAATGCTGATGCAGGTACAG‐3′	

### 2.13. Statistical Analysis

Data were analyzed using OriginPro software (Northampton, Massachusetts, USA). Results are presented as mean ± standard error of the mean (SEM). To determine the size distributions of MNPs, ImageJ software was used to measure the diameters of more than 70 individual MNPs per sample. The average sizes are reported as mean ± standard deviation (SD). The normality of the data has been assessed using the Shapiro–Wilk and Kolmogorov–Smirnov tests. Outliers were removed using the Grubbs test. For datasets that met the assumption of normality, one‐way analysis of variance (ANOVA) was performed. The homogeneity of variances was examined using the Levene test, and, based on the result, the Welch ANOVA or the Tukey or Bonferroni post hoc test was applied. The *p*‐values < 0.05 were considered statistically significant (ns: not significant, ^∗^ *p* < 0.05, ^∗∗^ *p* < 0.01, ^∗∗∗^ *p* < 0.001, and ^∗∗∗∗^ *p* < 0.0001).

## 3. Results and Discussion

### 3.1. High Crystallinity and Polydopamine‐Coated MNPs Were Produced in a Single Step

Surface modification of Fe_3_O_4_ MNPs with polymers is challenging due to the need for additional purification and reaction steps [24]. A one‐pot synthesis of MNPs with high crystallinity and surface modification with the desired molecule in a single step is valuable for large‐scale synthesis in biomedical and industrial applications. In this study, MNP1 and MNP2 were produced via a modified co‐precipitation method for potential use in cancer therapy. The co‐precipitation method was preferred due to its advantages, such as low cost, not requiring toxic chemicals, and high yield [4, 7]. In this method, iron salts (such as ferric chloride or ferrous sulfate) are dissolved in aqueous solution, and the metal ions are reduced by adding a base (e.g., ammonia or NaOH). The type of base affects the reaction pH and, in turn, MNP growth. In alkaline conditions, DA undergoes self‐polymerization and forms a layer on the surface of Fe_3_O_4_ MNPs [25, 26]. This study investigates whether standard co‐precipitation conditions enable simultaneous DA polymerization and PDA‐Fe_3_O_4_ MNP formation using different base sources. In addition, the anticancer potential of the resulting nanomaterials is evaluated.

**FIGURE 1:**
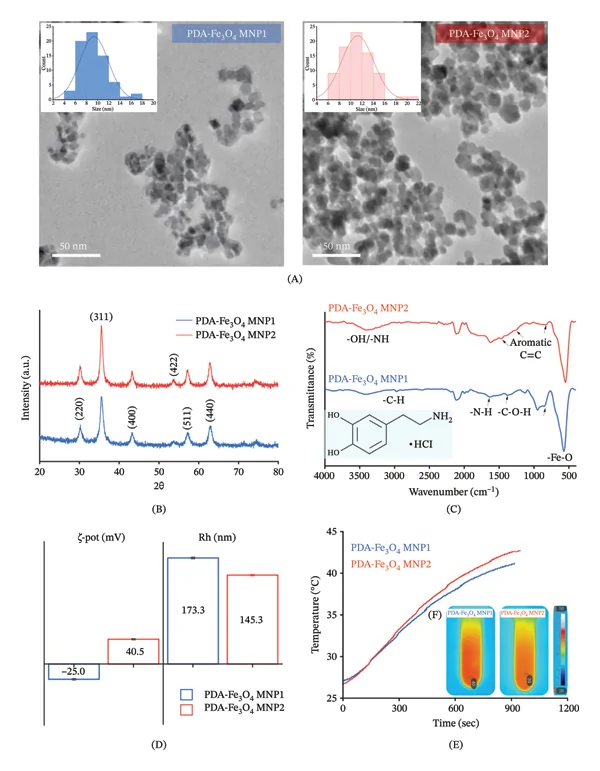
Physicochemical characterization of PDA‐Fe_3_O_4_ MNPs. (A) TEM images and histograms of MNPs, (B) XRD pattern, (C) FTIR spectrum (arrows indicating the characteristic PDA peaks on MNPs spectra), (D) DLS results, (E) temperature‐time graph of MNPs solutions (1000 ppm Fe) under AC magnetic field, and (F) thermal camera images of PDA‐Fe_3_O_4_ MNPs.

Both MNP1 and MNP2 were successfully synthesized and dispersed in water, with iron concentrations determined by ICP‐MS as 1.66 × 10^4^ ppm and 1.55 × 10^4^ ppm, respectively. The size and shape of the MNPs were examined in detail using TEM (Figure 1A). The SAR value of the MNP was calculated to be around 100 W/g at an applied field of 250 kHz. SAR values for iron oxide MNPs vary widely in the literature (tens to hundreds of W/g), depending on particle size, morphology, surface, and field conditions [27]. All synthesized MNPs were uniform and nearly spherical. The dimensions of MNPs were 9.36 ± 2.59 nm for MNP1 and 11.24 ± 2.82 nm for MNP2, determined from TEM images. No aggregation was observed in the micrographs, indicating satisfactory colloidal stability and homogeneous dispersion of the MNPs in water. The PDA shell was observed in the images of the products obtained in the NaOH‐based synthesis, but prominent shell was observed in the synthesis with ammonia. Notably, this observation may explain the observed difference in MNP sizes. PDA depositions may have prevented the growth of MNP by preventing new Fe‐O interaction [24].

The XRD pattern of the synthesized MNPs corresponds with the literature on magnetite [12, 28] (Figure 1B). The peaks corresponded to the (220), (311), (400), (422), (511), and (440) crystallographic planes, with 2‐theta values of 30°, 35°, 43°, 53°, 57°, and 62°, respectively. The peak values are similar to the characteristic XRD peaks of the magnetite, which has a cubic spinel structure. The sharp peaks reveal the magnetite’s high crystalline structure. The sizes of MNPs determined using the Scherrer equation were 6.98 nm and 10.39 nm for MNP1 and MNP2, respectively, which closely match the sizes observed in TEM, indicating uniform particle sizes.

FTIR spectroscopy was used to determine the molecular structures on the surface of MNPs (Figure 1C). The broad peak attributed to amine and hydroxyl reduction was observed at 3397 cm^−1^ and 3407 cm^−1^ for MNP1 and MNP2, respectively [29, 30]. The characteristic peak for PDA corresponding to the bending vibration of the N‐H bond was seen at 1623–1638 cm^−1^, proving the deposition of a PDA shell on the surface of the MNPs [31]. The FTIR spectra of MNP2 exhibited distinct peaks at 1252 and 1468 cm^−1^, corresponding to PDA’s C‐O and C=C bonds, respectively. These peaks are less prominent in MNP1. The range of 700–800 cm^−1^ indicates the presence of aromatic C‐H bonds[32]. The absorption band between 1500 and 1700 cm^−1^ reveals a carbonyl stretching vibration, indicating that PDA has successfully coated on Fe_3_O_4_ MNPs [33]. The PDA‐related peaks are more pronounced in the MNP1 sample. This finding further confirms that a shell surrounds MNP1, as observed in TEM micrographs. The Fe‐O bond was detected at 567 cm^−1^ and 543 cm^−1^ in MNP1 and MNP2, respectively. The presence of the polymer and the reactive base source shifted the Fe‐O peak in the structure. All FTIR data are consistent with previously published works [26, 32]. The FTIR spectrum reveals that DA polymerization was successfully achieved by one‐pot synthesis. Polymerized DA is usually prepared by self‐polymerization of DA in a weakly alkaline state, and when iron ions are included in this environment, the structure can acquire magnetic properties [34].

The PDA‐Fe_3_O_4_ MNPs exhibited excellent colloidal stability and dispersion properties in aqueous solutions (Figure 1D). DLS results revealed that the Rh of MNP1 and MNP2 were ∼173.3 nm and 145.3 nm, while the PDIs were 0.254 and 0.183, respectively. Although TEM and XRD analyses exhibited that MNP2 possesses a larger core size than MNP1, DLS measurements showed the opposite trend. This indicates that the PDA shell observed on the surface of MNP1 in TEM images contributes substantially to its hydrodynamic diameter by increasing not only the apparent particle size but also the thickness of the hydration layer surrounding the polymer‐coated surface. Interestingly, the base source used in the synthesis step significantly affects the MNP surface charge. In MNP1, the ζ‐potential value was −25.0 mV, while in MNP2, it was +45.5 mV. The noticeable difference in zeta potential between MNP1 and MNP2 indicates that the choice of base affects not only the surface functional groups but also the polymerization efficiency. It is known that DA is a positively charged compound. In our study, self‐polymerization of DA on MNPs in a basic solution was expected. However, during DA binding to the MNPs, the surface charge may shift to negative values due to DA’s amphiphilic properties, depending on the environmental pH. Similar observations have been reported in the literature [35, 36]. Some studies report that DA‐modified nanoparticles have a negative zeta potential [37], and another study shows that the surface zeta potential of superparamagnetic Fe_3_O_4_ MNP decreased from +25.78 mV to +13.92 mV after DA functionalization [38]. These findings indicate that DA reduces the positive charge density on the surface by imparting a negative charge to the Fe_3_O_4_ surface. The results of these studies align directionally with our findings.

Nevertheless, MNP aqueous solutions at low concentrations (1000 ppm Fe) reached 40°C in approximately 10 min under an alternating current (AC) magnetic field (Figure 1E). Thermal camera photos after removing the tubes from the device supported these findings (Figure 1F). According to our research, the temperature rises of MNP solutions maintained under an AC magnetic field did not correlate with the base source or the surface coating molecules. The ability of MNPs to kill cancer cells by heating them in a biological environment depends on several factors, including the coating material, the medium density, and the particle cluster. MNPs generate heat through three mechanisms: the Néel mechanism, which is based on the magnetic moment fluctuations within the particle; the Brown mechanism, which is based on the particle’s physical motion; and, in larger particles, heat generation due to magnetic field losses (hysteresis) [39]. The amount of heat generated is measured by SAR. SAR depends on the particle’s properties, the strength/frequency of the magnetic field, and how the particle interacts with its environment. Different coating materials on MNPs (e.g., SiO_2_) or surface modifications of MNPs can retain heat and reduce SAR. Also, a broader size distribution (with particles of varying sizes) reduces overall heating efficiency [40].

To our knowledge, this is among the first studies to systematically compare the thermo‐oxidative stress profiles of two PDA‐Fe_3_O_4_ MNP variants synthesized using different alkali sources (NaOH vs. NH_4_OH), which yield distinct physicochemical characteristics. Consequently, the base source used in the synthesis affects the size, morphology, surface charge, and stability of PDA‐coated Fe_3_O_4_ MNPs, but does not significantly affect the MNPs’ heating ability.

### 3.2. Internalization and Dose‐Dependent Cytotoxic Effects of PDA‐Fe_3_O_4_ MNPs on HUVEC and SH‐SY5Y Cells

The literature indicates that bare Fe_3_O_4_ nanoparticles exhibit rapid aggregation, loss of colloidal stability, and irregular sedimentation due to their high surface energy [41]. This causes an artificial increase in cell–surface interactions, thereby distorting the true dose–response relationship. This situation may lead to the observed toxicity arising from physical accumulation and local concentration rather than from the nanoparticle’s chemical effects. In contrast, PEG, dextran, or polymer coatings increase the dispersibility of nanoparticles, ensuring their stability in the environment, balancing protein corona formation, and enabling more controlled cellular uptake [41, 42]. Additionally, coating enhances the system’s biocompatibility by reducing iron‐ion release and ROS production and by improving reproducibility across experiments.

Monomeric DA has multiple functions in the brain and serves as an endogenous ligand for DA receptors [43]. PDA, employed in biological applications, is produced by the oxidative polymerization of monomeric DA in alkaline solutions. PDA is a remarkable material due to its biocompatibility, surface adhesiveness that enhances cellular uptake of cargo molecules, and its ability to facilitate the effortless immobilization of biomolecules. On the other hand, DA serves as an SH‐SY5Y cell‐targeting molecule [44]. DA‐modified MNPs can be taken up more readily by SH‐SY5Y cells, potentially enhancing their therapeutic effect.

In this study, SH‐SY5Y human neuroblastoma cells were selected as the cancer cell model, and HUVECs were selected as the normal cell model, as used in many studies [45–47]. SH‐SY5Y cells were selected because they model neuroblastoma, a high‐risk pediatric cancer; they express key regulators of ferroptosis (glutathione peroxidase 4 [GPX4], SLC7A11, and ACSL4); and they are widely used to investigate the biological effects of iron‐based nanoparticles. HUVECs were used as a normal, noncancerous control cell line to evaluate the biocompatibility and cell‐type specificity of the nanoparticles. Additionally, endothelial cells provide a biologically meaningful system not only as a standard model for vascular toxicity studies but also for investigating processes related to tumor microvasculature.

**FIGURE 2:**
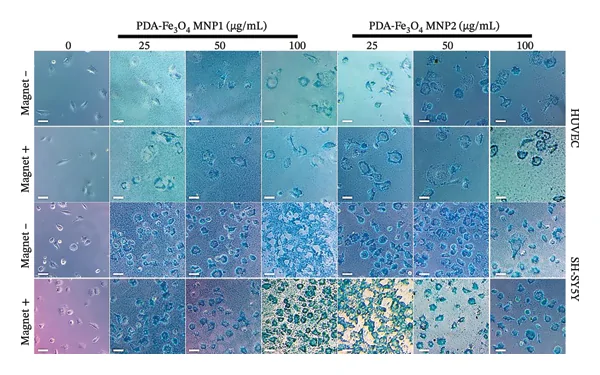
Intracellular uptake of PDA‐Fe_3_O_4_ MNPs into HUVEC and SH‐SY5Y cells. PDA‐Fe_3_O_4_ MNPs were detected by Prussian blue staining after incubation for 6 h. Cells were kept on a magnet for 10 min after PDA‐Fe_3_O_4_ MNP treatment in magnet+ groups—scale bar: 50 μm.

In this study, Prussian blue dye was used to evaluate the intracellular iron distribution of PDA‐Fe_3_O_4_ MNPs. Prussian Blue is a widely used histochemical method for visualizing iron‐based MNPs within cells [43]. According to the staining results, blue granules were detected in cells treated with PDA‐Fe_3_O_4_ MNP, whereas no granules were detected in the control groups. Cells internalize MNPs in a dose‐dependent manner, as demonstrated by the increase in granule density with increasing concentration (Figure 2). An increase in granule accumulation was observed with the application of a magnet. These findings support that magnetic guidance strategies could enhance the uptake of MNPs. Additionally, significant differences in localization patterns are observed depending on cell types. Blue precipitates in HUVECs are mostly confined to the membrane boundaries, whereas in SH‐SY5Y cells, they are dispersed throughout the cytoplasm. Additionally, the more intense accumulation of MNP2, which has a positive surface charge, on the membrane in both cell types indicates that electrostatic interactions strengthen the interaction between the cell surface and the MNPs. However, total internalization was higher in SH‐SY5Y cells than in HUVECs, which may be related to DA’s potential biochemical interactions with neuronal cells.

**FIGURE 3:**
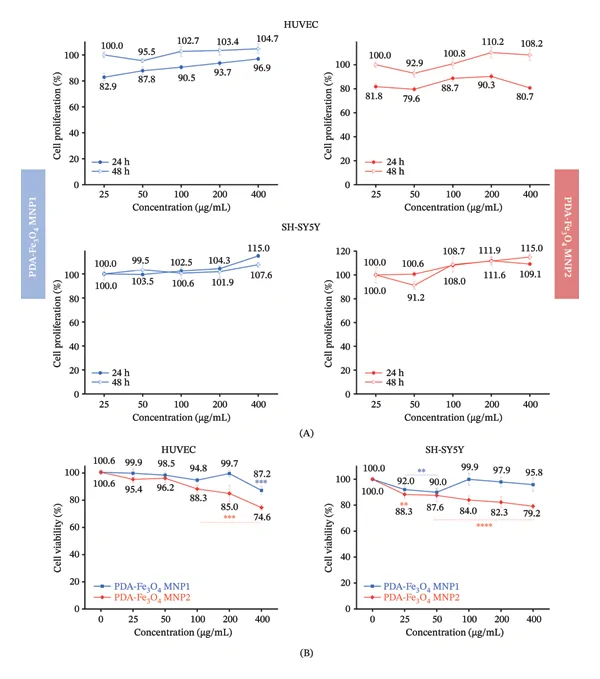
Cytotoxic effects of PDA‐Fe_3_O_4_ MNPs on HUVEC and SH‐SY5Y cells. (A) Proliferation rates of the cells under treatments of PDA‐Fe_3_O_4_ MNPs for 24 and 48 h, as revealed by crystal violet assays. (B) Cell viability of the cells under treatments of PDA‐Fe_3_O_4_ MNPs for 48 h, as revealed by MTS assays. Statistical analyses were performed using ANOVA. Values in the graph represent mean ± SE (*n* > 3).

The effects of PDA‐Fe_3_O_4_ MNPs on proliferation in HUVEC and SH‐SY5Y cells were examined at different doses during 24‐ and 48‐h incubation periods. The percentage proliferation values obtained from the CV test indicated that the cells remained viable and retained adhesion capacity. CV staining is a well‐established method for evaluating cell adherence, colony formation, and proliferation in culture flasks [13]. The proliferative activity of the PDA‐Fe_3_O_4_ MNPs‐treated HUVEC and SH‐SY5Y was determined after 24 and 48 h (Figure 3A). It is believed that the ability of MNPs to stimulate cell growth in HUVECs after 48 h of incubation is mediated by multiple biological mechanisms. DA present on the surface of PDA‐Fe_3_O_4_ MNPs may have promoted proliferation by activating growth‐factor‐like signals in cells [48]. Additionally, the surface chemistry of MNPs may enhance cell proliferation by enabling stronger cell adhesion [38, 49]. The literature indicates that PDA nanoparticles effectively scavenge ROS and reduce oxidative stress, thereby supporting healthy cell proliferation [50]. Ultimately, increased iron levels may activate ferritin‐mediated protective metabolic pathways in cells, thereby enhancing their ability to adapt to adverse conditions and promoting cell proliferation [51]. In summary, the higher proliferation rate at 48 h compared to 24 h may be attributed to the increased accumulation of MNPs inside or on the surface of the cells over time, cellular adaptation to MNP exposure, the proliferative effects of the PDA surface coating, and the natural growth dynamics associated with the cell cycle.

The MTS assay assessed the cytotoxicity of PDA‐Fe_3_O_4_ MNPs on HUVEC and SH‐SY5Y cells by measuring intracellular metabolic activity (Figure 3B). A dose‐dependent reduction in metabolic activity was observed in both cell lines, indicating that PDA‐Fe_3_O_4_ MNPs remain biocompatible up to 200 μg/mL. MNP1 demonstrated minimal impact on both cell types. MNP1 treatment at 400 μg/mL in HUVECs resulted in 87.2% cell viability. MNP2, on the other hand, exhibited more pronounced dose‐dependent cytotoxicity in HUVECs; viability was largely preserved at low doses (95.4%–96.2%), while cell viability decreased to 74.6% at 400 μg/mL. Cell viability was dose‐dependently reduced in the MNP2‐treated cells. This is expected, as MNP1 has lower uptake rates than MNP2. These results indicate that MNP types exhibit minimal toxicity in healthy endothelial cells at low doses (up to 100 μg/mL), supporting their potential biocompatibility. In SH‐SY5Y cells, MNP1 showed limited effects, maintaining viability above 90% in the 25–400 μg/mL concentrations. In contrast, MNP2 caused a dose‐dependent decrease in rate, from 88.3% at 25 μg/mL to 79.2% at 400 μg/mL. A dose of 50 μg/mL was selected for subsequent experiments based on its subtoxic but biologically active profile in both cell lines.

In conclusion, the efficient internalization and dose‐dependent cytotoxic effects of PDA‐Fe_3_O_4_ MNPs demonstrate their potential as targeted therapeutic agents for brain cancer. Studies on these issues contribute to a better understanding of the role of DA in neuronal health and the pathophysiology of diseases [52]. In the literature, a dose‐dependent effect was reported in SH‐SY5Y cells treated with DA (IC_50_:202 μM) [53]. In our study, IC_50_ values could not be determined within the tested concentration range. However, DA’s relationship with cancer is a complex issue, and its effects on cancer biology have not been fully understood.

### 3.3. Effect of MHT

Factors such as size, shape, surface coating, and magnetic material affect the heating capacity of MNPs under an AC magnetic field. This property, which also reveals the magnetic properties of MNPs, is defined as MHT [54]. The magnetism of MNPs may be modified when organic molecules, such as polymers, are coated on them [55]. The published studies indicated that complex interactions between surface coating and magnetic properties are not understood [56].

**FIGURE 4:**
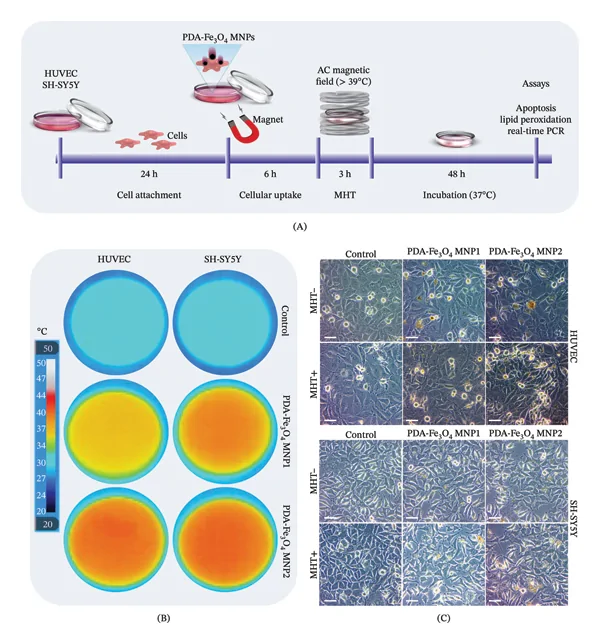
Magnetic hyperthermia (MHT) application. (A) Schematic illustration of treatment steps. The figure shows HUVEC and SH‐SY5Y cells exposed to PDA‐Fe_3_O_4_ MNPs after a 24‐h adhesion period, with cellular uptake facilitated by a magnet (6 h). The cells were then exposed to a 3‐h MHT treatment under an alternating magnetic field (> 39°C), followed by a 48‐h incubation, after which analyses for apoptosis, lipid peroxidation, and real‐time PCR were performed. (B) Thermal camera (immediately after exposure to a magnetic field) and (C) inverted microscope images (48 h) of HUVEC and SH‐SY5Y after treatments. The scale bar is 50 μm. PDA‐Fe_3_O_4_ MNPs were treated at a 50 μg/mL concentration.

The cells were exposed to PDA‐Fe_3_O_4_ MNPs and an AC magnetic field for 3 h (Figure 4A). The temperature of the cell culture plates increased due to the magnetic field. The scale in the thermal camera images is set to a 3°C difference (Figure 4B). These images show that the cell temperature did not exceed 41°C during magnetic field application. Thermal images of culture dishes showed a greater temperature increase in MNP1‐treated SH‐SY5Y wells compared to HUVEC wells. These results may reflect differences in MNP internalization rather than intracellular temperature changes. This phenomenon might be attributed to the target specificity driven by the negative zeta potential of MNP1 (Figure 1D) and the high amount of DA receptors in SH‐SY5Y [52]. The better heating of MNP2‐treated cells may be due to higher MNP internalization, as MNP2’s surface is positive. This suggests that MNP2 could potentially contribute to effective MHT‐assisted cancer therapy. Lastly, while minimal morphological changes were observed in MNPs + MHT‐treated HUVECs (spherical shape), significant alterations in cell morphology were detected in SH‐SY5Y cells (intercellular junctions appeared loosened, and distinct cellular extensions were formed) under the same treatments (Figure 4C).

**FIGURE 5:**
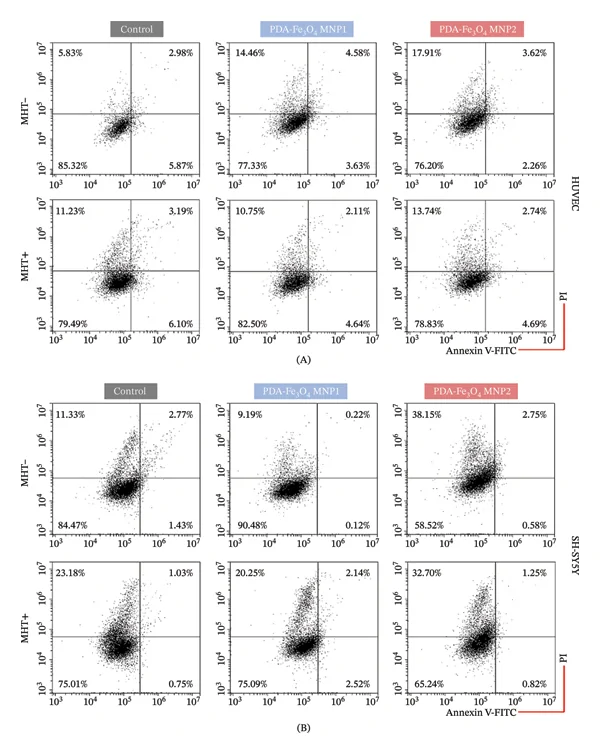
Apoptosis results of MNPs‐ and MHT‐treated cells. (A) HUVEC and (B) SH‐SY5Y cells were incubated for 48 h before the test.

Cellular responses induced by PDA‐coated MNPs (MNP1 and MNP2) in HUVEC and SH‐SY5Y cells were evaluated both before (MHT−) and under MHT (MHT+). In the control groups, the percentage of viable cells was high (> 84%) in both cell lines, and the rates of early and late apoptosis were relatively low (< 10%) (Figure 5A,B). Under MHT (MHT+), the combination of MNP2 + MHT significantly increased cell death in both cell lines (viability is 78.83% and 65.24% for HUVEC and SH‐SY5Y, respectively). HUVECs were generally more resistant to MNPs and hyperthermia, and the proportion of viable cells remained higher than that of SH‐SY5Y cells. In contrast, SH‐SY5Y cells showed higher PI positivity, particularly in the MNP2 + MHT condition (> 33%), suggesting that neuronal cells are more sensitive to thermal and oxidative stress. The cell viability is reduced in the SH‐SY5Y cells treated with MNP2 and MNP2 + MHT, and the percentage of PI+ cells is raised (40.8% and 33.9%, respectively) (Figure 5B). Remarkably, the therapeutic effect of MHT was observed when MNP1 was administered, whereas this effect was not observed in MNP2 treatments in SH‐SY5Y. In HUVECs, viability never declined below 75%, reflecting their higher tolerance to both MNP exposure and thermal stress. The increase in total cell death under MHT+ conditions was primarily attributed to cells in the PI+, characterized by impaired membrane integrity, while early apoptosis rates remained low (< 3%). This finding suggests that local heating induced by magnetic fields primarily disrupts membrane integrity rather than activating classical apoptotic pathways, as reported in the literature [57]. Supporting this interpretation, our Prussian Blue staining results (Figure 2) revealed blue granules attached to the cell membrane, indicating that MNP2 may have compromised membrane integrity and facilitated PI penetration. Consistent with this observation, Annexin V staining did not show increased levels of apoptotic markers. Instead, a marked rise in PI‐positive cells—reflecting loss of membrane integrity and non‐apoptotic cell death—was detected. The increase in PI‐positive cells may be attributed to loss of membrane integrity caused by MHT or to non‐apoptotic cell death pathways, such as ferroptosis [58, 59]. This increase may be due to localized thermal effects, including subcellular temperature increases during AC magnetic field exposure, as shown by the surface thermal imaging in Figure 4B. Furthermore, the observed lipid peroxidation and ROS profiles suggest that oxidative damage associated with ferroptosis, rather than apoptosis, may also contribute to this process. Both mechanisms are biological possibilities that could explain membrane destabilization. However, consider that MNPs can intrinsically enhance membrane permeability, directly correlating with the increase in PI+ cells. Therefore, additional analyses were performed to clarify the mechanisms underlying cell death.

**FIGURE 6:**
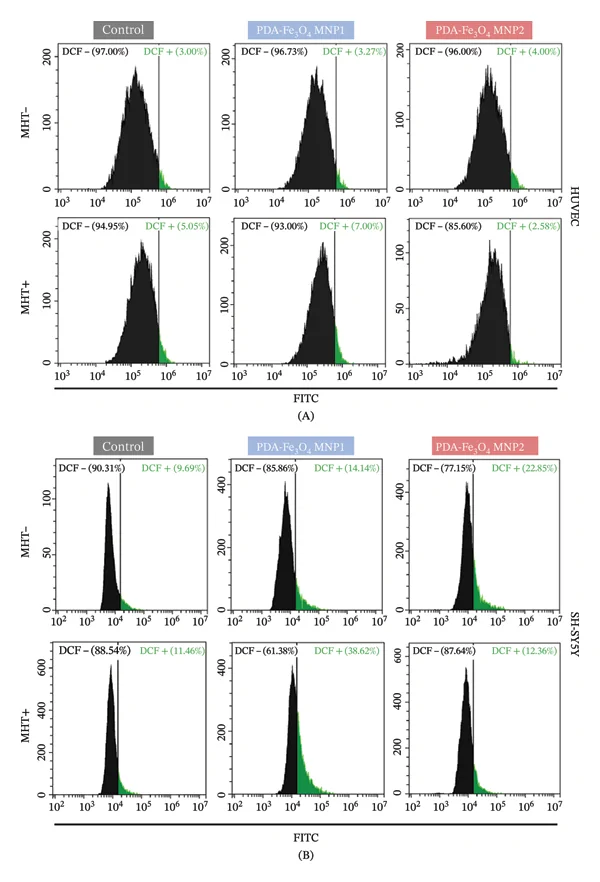
ROS results of MNPs‐ and MHT‐treated cells. (A) HUVEC and (B) SH‐SY5Y cells were incubated for 48 h before the test.

In the ROS measurements, low levels of DCF positivity were observed in both cell lines within the control groups. MNPs did not show oxidative stress in HUVECs, either MHT− or MHT+ (Figure 6A). The DCF+ rate was slightly increased in the MNP1 + MHT group (7%), indicating that MNP1 might induce thermal‐oxidative stress by providing more effective heating in the magnetic field. In the MHT− group, both MNP1 and MNP2 administration increased ROS levels, with the increase particularly pronounced in the MNP2 group, especially in SH‐SY5Y (Figure 6B). Responses varied by cell type and MNP type under MHT+ conditions. A significant increase in ROS was observed in the MNP1 + MHT (38.62%), supporting the thermal‐oxidative stress capacity of MNP1. A 22.85% DCF+ cell population was observed in MNP2‐treated SH‐SY5Y cells. This is higher than that of MNP2 + MHT (12.36%).

The elevated ROS levels observed under MNP1 + MHT conditions can be attributed to the combined effects of Fe^2+^‐mediated Fenton reactions and the relatively weaker antioxidant protection afforded by its thin PDA coating. Although TEM and FTIR analyses did not reveal a prominent PDA shell around MNP2, its markedly positive zeta potential (+45.5 mV) suggests that a thin, incompletely polymerized dopamine layer may nonetheless be present on its surface—sufficient to alter surface charge without forming an optically resolvable coating. Given that ROS are highly reactive and rapidly interact with membrane lipids, their accumulation promotes lipid peroxidation, a key event in ferroptosis. Notably, DA‐based systems may exhibit concentration‐dependent dual behavior, acting as pro‐oxidants at low DA‐to‐iron ratios and exerting protective effects at higher ratios by modulating iron homeostasis [50, 60–63]. Moreover, both DA‐mediated iron release from ferritin and hyperthermia‐induced iron release from MNPs may contribute to increased ROS production and lipid peroxidation. Collectively, these findings suggest that MHT‐driven enhancement of ROS production and lipid peroxidation can overcome cellular antioxidant defenses, leading to ferroptosis‐associated cell death. Overall, MNP1 appears to potentiate thermo‐oxidative stress, whereas MNP2 may induce a cellular response more related to membrane integrity and redox buffering, reflecting differences in their intracellular interactions and thermal behavior.

**FIGURE 7:**
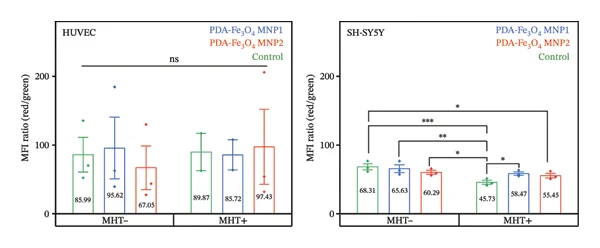
Lipid peroxidation assay results of MNPs‐ and MHT‐treated cells. Comparison of the red channel (PE) signal intensity with the green channel (FITC) signal intensity in the flow cytometry measurements determined the result. MFI: mean fluorescence intensity. The red/green MFI ratio decreases under conditions of increased lipid peroxidation.

Lipid peroxidation is the primary determinant of the iron‐dependent cell death mechanism known as ferroptosis, characterized by ROS‐induced oxidative degradation of polyunsaturated fatty acids in the cell membrane [63, 64]. The degree of lipid peroxidation is directly related to the DA/iron ratio, indicating DA’s potential to both promote and prevent oxidative damage [63]. Here, lipid peroxidation levels were assessed using a ratio‐metric PE/FITC fluorescence sensor, which shifts its emission from red to green upon ROS‐induced oxidation (Figure 7). In brief, low red/green ratios are associated with increased lipid peroxidation, thereby increasing the risk of ferroptosis. These values were consistently above 85% in HUVECs, except when MNP2 was applied. Statistical analyses showed no significant differences between the application groups in HUVECs; these findings suggest that ferroptosis‐associated cell death does not occur in these cells.

In SH‐SY5Y cells, a significant decrease in the red/green ratio was observed in the MHT+ groups compared to the MHT− groups. This finding indicates a trend consistent with ferroptosis stress under MHT treatment in SH‐SY5Y cells. When MHT was not applied, the red/green ratio was ∼65 in the MNP1 application, whereas it decreased to ∼58 under MHT+ conditions. Similarly, in the MNP2 application, MHT reduced the red/green ratio from ∼60 (MHT−) to ∼55 (MHT+). Interestingly, this decline is less than that observed with MHT. The red/green ratio was highest (∼68) in the control group that did not perform MHT, indicating low lipid peroxidation. The significant decrease in the red/green ratio (∼46) in the control group after MHT application indicates enhanced lipid peroxidation, consistent with our gene expression findings that magnetic field exposure alone can induce oxidative stress‐related transcriptional changes in SH‐SY5Y cells even without exogenous nanoparticles. This result indicates that MHT alone can promote lipid peroxidation, likely through the catalytic effect of intracellular (endogenous) iron ions, and that this baseline effect may be modulated by the presence of a PDA coating in the MNP‐treated groups.

This finding suggests that the PDA coating of MNP may attenuate MHT‐induced ferroptosis‐associated cell death through its antioxidant and radical‐scavenging properties. Indeed, PDA‐coated MNPs of different dimensions exhibit dose‐ and time‐dependent oxidative stress, with a notable apoptotic effect in cells [31]. There are conflicting findings in the literature regarding whether a static magnetic field can increase ROS production, with effects varying by oxidant type, magnetic field intensity, exposure time, and cell type [65]. In a study investigating the effects of radiofrequency electromagnetic fields modulated at specific frequencies, very low levels of radiofrequency electromagnetic fields were shown to inhibit the growth of tumor cells at specific modulation frequencies [66]. It has been reported that applying a magnetic field to cancer cells, including G401, A549, SGC‐7901, and PANC‐1, significantly inhibits their growth while increasing ROS production and lipid peroxidation [2]. Therefore, the coating thickness, surface chemistry, and formulation optimization of PDA‐Fe_3_O_4_ MNPs should be reevaluated for ferroptosis‐based therapeutic approaches targeted at cancer cells.

As a result, the MNP1+MHT group, which showed the largest increase in ROS, also exhibited the highest levels of ferroptosis in the lipid peroxidation assay. This finding suggests that formulations such as MNP1, which have limited protective effects against PDA, may be more suitable candidates for ferroptosis hyperthermia treatments.

### 3.4. Gene Expression Levels of MNPs‐ and MHT‐Treated Cell Lines

qRT‐PCR analysis was conducted to identify gene expression changes in HUVEC and SH‐SY5Y cells following exposure to MNPs and MHT. Significant changes in gene expression were observed in SH‐SY5Y cells exposed to only magnetic fields (in the MHT control groups). This may be attributable to the magnetic field’s effect on altering cellular signal transduction pathways by (i) affecting ion channels or receptors in the cell membrane, inducing oxidative stress, or affecting critical signaling pathways within the cell; (ii) epigenetic changes leading to DNA methylation or histone modifications; or (iii) affecting the cell membrane or cytoskeleton to alter the expression of genes related to cell shape and division [67].

**FIGURE 8:**
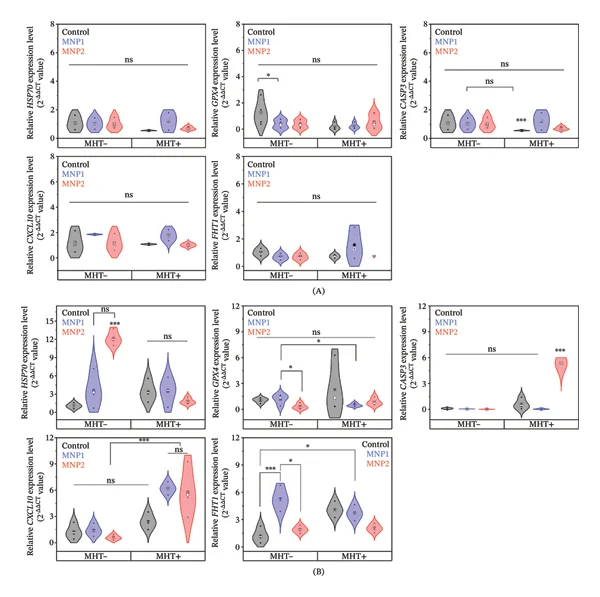
Gene expression levels in PDA‐Fe_3_O_4_ MNPs‐treated cells. (A) HUVEC and (B) SH‐SY5Y cells. Gene expression changes determined by the 2^−ΔΔCT^ value were normalized to *GAPDH* and nontreated control cells (*n* = 3). *HSP70*: heat shock protein 70 (or DNAK), *GPX4*: glutathione peroxidase 4, *CASP3*: caspase‐3, *CXCL10*: C‐X‐C motif chemokine ligand 10, also known *IP-10*, and *FTH1*: ferritin heavy chain. In the violin plots, squares denote the mean values and triangles indicate the median values.

Gene expression results from HUVECs indicate that administration of MNP1 and MNP2, either alone or in combination with MHT, causes a limited molecular response in endothelial cells (Figure 8A). The absence of significant changes in *HSP70*, *GPX4*, *CXCL10*, and ferritin heavy chain 1 (*FTH1*) gene expression compared with the control group suggests that exposure to MNPs does not significantly affect the cellular stress response, oxidative defense mechanisms, inflammatory signaling, or iron homeostasis. In particular, the absence of changes in *HSP70* expression indicates that the classical heat shock response was not activated despite MHT application, while the stability of *GPX4* and *FTH1* levels suggests that the cells were not under ferroptotic stress or iron‐induced oxidative stress (ns, *p* > 0.05). Similarly, the absence of a significant difference in *CXCL10* expression supports the finding that MNPs do not induce a significant proinflammatory effect in endothelial cells.

HSP70 is a heat shock protein that plays a role in cellular stress response [68]. *HSP70* gene expression in SH‐SY5Y cells (Figure 8B) showed highly statistically significant differences between groups (*p* < 0.0001). In cells treated with MNP2 without MHT (MHT−), *HSP70* expression increased 12.17‐fold compared to the control (MHT−). A significant increase was also observed under MNP1 conditions; both findings indicate that MNPs alone trigger a strong cellular stress response in neural cells (*p* < 0.0001). The suppression of *HSP70* under MHT+ conditions suggests that thermal stress has overwhelmed the cells’ adaptive capacity, or alternatively, that cells with high HSP70 expression were selectively eliminated by MHT‐induced cell death. Future studies using time‐course HSP70 measurements could distinguish these possibilities.

*GPX4* gene expression decreased (not statistically different) in MNP and MHT combination groups, indicating that the cells have insufficient capacity to cope with oxidative stress in SH‐SY5Y. A decrease in *GPX4* (an antioxidant defense gene) expression has been linked to ferroptosis, a cell death driven by lipid peroxidation. As a glutathione‐dependent selenoenzyme, *GPX4* protects against ferroptosis by reducing toxic lipid hydroperoxides to harmless lipid alcohols. The suppression of *GPX4* activity leads to excessive accumulation of lipid peroxides in cells and promotes ferroptosis [69]. Although the decrease in *GPX4* expression did not reach the statistical significance level, the observed downward trend suggests that SH‐SY5Y cells have a limited capacity to cope with oxidative stress. The observation of high lipid peroxidation levels (Figure 7), together with low GPX4 levels in the MNP + MHT group, supports this mechanism.

Caspase‐3 (CASP3) is a protease that initiates apoptosis by cleaving intracellular substrates. In our study, significant differences in *CASP3* gene expression wasobserved between MNP2 + MHT and all other groups (*p* < 0.0001) in SH‐SY5Y. The low *CASP3* expression across all groups is consistent with the low Annexin V positivity in the flow cytometry results (Figure 5). Studies have focused on developing apoptosis tests that detect *CASP3* [70]. The absence of *TP53* expression in all groups (data not shown) suggests that *CASP3* expression may proceed via a pathway distinct from the classical TP53‐dependent apoptotic cascade. The literature indicates that *CASP3* activity at sublethal or nonlethal levels can regulate vital processes, including cell cycle progression, proliferation, differentiation, and even cancer progression, independently of apoptosis [71]. Considering these findings, the dual role of *CASP3* in cell fate underscores the importance of considering both its apoptotic and non‐apoptotic functions when evaluating the efficacy of therapeutic strategies.

*CXCL10* gene expression associated with the inflammatory response was higher (*p* < 0.0001) in the MHT‐treated SH‐SY5Y cells (Figure 8B). The noticeable increase in *CXCL10* expression in SH‐SY5Y cells following MHT treatment suggests that this chemokine coordinates an inflammatory response in neural cells under MNP‐mediated thermal stress. It has been demonstrated that neuronal apoptosis is triggered by caspase‐3 activation in neural cultures exposed to *CXCL10* and that *CXCL10* and *CASP3* are co‐expressed in the same cells. In our study, concurrent increases in *CXCL10* and *CASP3* in the MNP2+MHT+ group support a cascade relationship between these two pathways. On the other hand, it should not be overlooked that MHT treatment may support immune activation and, thereby, contribute to long‐term cancer therapies by activating the immune system’s memory mechanism.

In SH‐SY5Y cells, *FTH1* gene expression, the main intracellular iron storage protein, increased in all groups. This may suggest that iron uptake is more prominent in SH‐SY5Y cells. A significant increase (5.2‐fold) in *FTH1* (iron homeostasis) gene expression was observed with MNP1 treatment (*p* < 0.0001), suggesting an iron imbalance or increased iron load in the SH‐SY5Y cell. The increase in FTH1 clearly demonstrates that it activates the intracellular iron‐buffering capacity of neuronal cells against the free‐iron load derived from MNPs. Ferritin, composed of FTH1 and FTL1 subunits, is the primary intracellular iron storage protein [72, 73]. Elevated FTH1 expression likely reflects a cellular iron‐buffering response to MNP‐derived iron load. Also, NCOA4‐mediated ferritinophagy—the autophagic degradation of ferritin—can release sequestered iron and thereby promote ferroptosis [72]. Under normal conditions, iron‐induced oxidative stress is prevented by *FTH1* by storing free iron ions (Fe^2+^/Fe^3+^). However, if *FTH1* expression is low, there is an uncontrolled increase in intracellular iron ions, and consequent ROS formation occurs. This step is especially critical in the induction of iron‐dependent programmed cell death, known as ferroptosis. Thus, *FTH1* expression supports MNP’s regulatory effect on cell death. At the gene expression level, genes such as *FTH1*, *GPX4*, and *SLC7A11* determine cells’ susceptibility to ferroptosis by regulating iron homeostasis and oxidative stress [73, 74]. In this context, the increase in *FTH1* observed in SH‐SY5Y cells may reflect an adequate cellular response to iron overload rather than direct induction of ferroptosis; nevertheless, *GPX4* data also suggest that this defense mechanism becomes insufficient once a certain threshold is exceeded.

When the findings observed in SH‐SY5Y cells are evaluated collectivelly, it appears that the combination of PDA‐Fe_3_O_4_ MNPs and MHT activates multiple stress and cell death pathways simultaneously in this cell line, including the heat shock response, inflammatory signaling (*CXCL10*), the ferroptotic process (*GPX4* decrease and *FTH1* increase), and apoptosis (*CASP3*). While this multifaceted activation is significant for therapeutic efficacy, it also requires careful interpretation of potential adverse effects on neural tissues. It is known that iron oxide MNPs can cross the blood–brain barrier and cause toxic effects in neuronal cells; therefore, the safety profile must be rigorously evaluated in central nervous system applications [75].

The expression differences observed between MNP1 and MNP2—particularly the 12.17‐fold difference in *HSP70* and the divergent profiles in *CASP3* and *CXCL10*—suggest that the surface properties, particle size, or PDA coating thickness of the two formulations significantly shape their biological interaction profiles. In future studies, in‐depth characterization of these two formulations and concurrent investigation of additional ferroptosis‐specific biomarkers (GSH levels, ACSL4, RSL3‐positive control, or ferrostatin‐1 rescue experiments) would be investigated.

Our study presents an original and timely contribution by introducing a one‐pot synthesis strategy for PDA‐Fe_3_O_4_ MNPs and systematically demonstrating how base‐dependent surface chemistry governs their physicochemical properties and biological responses. By comparing two MNP variants with distinct PDA characteristics, we reveal mechanistically distinct pathways of cytotoxicity, including thermo‐oxidative stress and ferroptosis‐associated processes. It is proposed that the cellular response observed in our study can be explained by increased thermo‐oxidative stress that overwhelms antioxidant defenses, thereby enhancing ROS production and lipid peroxidation and triggering ferroptosis‐associated cell death. The demonstrated selective behavior between cancerous and non‐cancerous cells further underscores the translational relevance of this system. Overall, this work provides a meaningful framework for the rational design of surface‐engineered MNPs in mechanism‐driven cancer therapy and highlights their potential for future applications.

## 4. Conclusion

This study reports the one‐pot synthesis and biological evaluation of PDA‐Fe_3_O_4_ MNPs for MHT. It indicates that these MNPs can be transformed into multifunctional structures suitable for biomedical applications. Comparative analysis of Fe_3_O_4_ MNP1 (NaOH) and Fe_3_O_4_ MNP2 (NH_4_OH) systems reveal that synthesis conditions are decisive in determining MNPs’ properties and biological responses. The smaller size (9.4 nm) and negative surface charge (−25.0 mV) of MNP1 were characterized by higher ROS production (38.6%) and significant lipid peroxidation. This was supported by a significant increase in *FTH1* expression (5.2‐fold), indicating activation of an iron‐buffering response consistent with ferroptosis‐associated iron stress. Confirmatory experiments at higher MNP concentrations, longer incubation times under a magnetic field, or with ferroptosis‐specific inhibitors (e.g., ferrostatin‐1) should provide further clarification. In contrast, the larger size (11.2 nm) and significant positive surface charge (+45.5 mV) of MNP2 led to increased cellular uptake, accompanied by lower ROS production (22.8%) and limited lipid peroxidation. Nevertheless, disruption of membrane integrity and internalization‐mediated cytotoxicity were prominent features of MNP2 treatment. Furthermore, the presence of a PDA coating (visible shell) in MNP1 and its absence in MNP2 clearly demonstrate the influence of synthesis conditions on surface chemistry and the role of surface chemistry in biological interactions. The use of a neuroblastoma cell line (SH‐SY5Y) and a noncancerous endothelial cell line (HUVEC) provides direct evidence of differential cellular selectivity, a critical parameter for therapeutic translation. It was observed that SH‐SY5Y cells internalized significantly more MNPs than HUVECs, and applying a magnetic field further enhanced this uptake. While both MNP types generally exhibited acceptable biocompatibility, cellular responses differed considerably between cell types. Consequently, it appears that MNP1 predominantly induces a thermo‐oxidative stress‐mediated ferroptotic cell death mechanism, whereas MNP2 primarily causes cytotoxicity through membrane damage and cellular internalization. In this study, the use of Fe_3_O_4_ and PDA, along with the application of a magnetic field, can complicate the deconvolution of cellular signaling pathways. Therefore, based on our findings, we prefer the use of low‐dose MNPs with short‐term magnetic field exposure to achieve more controlled and interpretable biological responses. In conclusion, these findings demonstrate that surface charge and coating strategies in nanoparticle design are critical parameters in guiding cellular fate.

## Funding

This study was supported by the Scientific Research Foundation of Atatürk University (FBA‐2024‐13463) and the Scientific and Technological Research Council of Turkey (TUBITAK, Türkiye Bilimsel ve Teknolojik Araştırma Kurumu) (120Z958).

## Conflicts of Interest

The authors declare no conflicts of interest.

## Data Availability

The data that support the findings of this study are available from the corresponding author upon reasonable request.
